# Guidance to detect, evaluate and prevent the problem of selective reporting in trial publications

**DOI:** 10.1186/1745-6215-14-S1-O91

**Published:** 2013-11-29

**Authors:** Kerry Dwan, Paula R Williamson, Carrol Gamble, Julian Higgins, Jonathan Sterne, Douglas G Altman, Mike Clarke, Jamie J Kirkham

**Affiliations:** 1 The University of Liverpool, Liverpool, UK; 2 The University of Bristol, Bristol, UK; 3 The University of Oxford, Oxford, UK; 4 Queen’s University, Belfast, UK

## Background

Many aspects of a trial may be incompletely reported, including the outcomes collected and the full set of analyses undertaken. Selective reporting bias occurs when the inclusion of outcomes or analyses in the report is based on the results. We review and summarise the empirical evidence from studies that have assessed the selective reporting of outcomes and analyses and provide guidance to trialists to help reduce this problem.

## Methods

Two systematic reviews of studies that have examined randomised trials for i) evidence for publication bias or selective reporting of *outcomes* and ii) evidence for selective reporting of *analyses*. An international collaboration of experts will be brought together in July to discuss the available evidence alongside current reporting guidance for trials. Recommendations are being produced with regards to raising the awareness and safeguarding trial publications against selective reporting.

## Results

From twenty studies, of which four were newly identified, the evidence in the first systematic review demonstrated an association between statistically significant results and publication. Statistically significant outcomes had a higher odds of being fully reported compared to non-significant outcomes (range of odds ratios: 2.2 to 4.7). A further seventeen studies consider aspects of selective reporting such as statistical analyses; subgroup analyses and composite outcomes.

## Conclusions

This work highlights the evidence of selective reporting and demonstrates the importance of pre-specifying outcomes, analyses and reporting strategies during the planning and design of a clinical trial, for the purposes of minimising bias when findings are reported.

